# Molecular and Genetic Biomarkers in Prostate Cancer Active Surveillance: Recent Developments and Future Perspectives

**DOI:** 10.3390/genes17010071

**Published:** 2026-01-06

**Authors:** Stephanie F. Smith, Robert D. Mills, Colin S. Cooper, Daniel S. Brewer

**Affiliations:** 1 Department of Metabolic Health, Norwich Medical School, Bob Champion Research and Education Building, University of East Anglia, Norwich NR4 7UQ, UK; robert.mills@nnuh.nhs.uk (R.D.M.); colin.cooper@uea.ac.uk (C.S.C.); 2 Department of Urology, Norfolk and Norwich University Hospitals, Colney Lane, Norwich NR4 7UY, UK

**Keywords:** active surveillance, biomarkers, prostate cancer, liquid biopsy, extracellular vesicles

## Abstract

Background/Objectives: Active surveillance (AS) has become the standard of care for many men with localised prostate cancer, aiming to avoid the overtreatment of indolent disease while maintaining oncological safety. Despite improvements in diagnostic techniques, misclassification at diagnosis and the limited ability to predict disease progression remain major challenges in AS. Novel molecular and genetic biomarkers, assessed through liquid biopsy approaches, offer the potential to refine patient selection and support risk-adapted monitoring in AS. Methods: We conducted a narrative review of biomarkers in the context of AS for prostate cancer, framing the discussion in terms of the challenges in AS and how biomarkers may address these. PubMed and Embase were searched for English-language peer-reviewed studies published between 2000 and 2025. International guidelines (AUA, EAU, NCCN, NICE) and reference lists were reviewed manually. Priority was given to large prospective cohorts, meta-analyses, and high-impact publications. Results: Blood-based assays such as PHI and the 4K score, urinary tests including ExoDx and SelectMDx, and the Prostate Urine Risk (PUR) signatures have all shown associations with disease progression or decisions to undergo earlier treatment. However, studies are often small, use surrogate endpoints, and lack validation in MRI-integrated cohorts. Biomarkers appear most informative in men with Gleason Grade 1 (GG1) disease, while evidence in GG2 cohorts is limited. Cost-effectiveness, heterogeneity of endpoints, and uncertainty in managing discordant biomarker and MRI results remain barriers to clinical adoption. Conclusions: Molecular and genetic biomarkers show promise for improving AS by reducing diagnostic misclassification and enhancing prediction of progression. Future research should define clinically relevant cut-offs, clarify integration with MRI, and evaluate longitudinal use. Demonstrating utility in contemporary cohorts could enable the development of biomarker-guided, personalised AS that maintains safety while minimising harm.

## 1. Introduction

Radical treatments for localised prostate cancer, such as surgery or radiotherapy, carry a substantial risk of long-term side effects, including urinary incontinence and erectile dysfunction, which can significantly impact patient quality of life [1,2]. Avoiding the overtreatment of indolent tumours is therefore a major priority in contemporary prostate cancer care [3]. Active surveillance (AS), a structured monitoring strategy intended to defer or avoid radical treatment in appropriately selected patients, has emerged as a key approach to address this issue [4].

The UK National Institute for Health and Care Excellence (NICE) guidelines (NG131) endorse AS as the first-line option for men with Cambridge Prognostic Group 1 (CPG1) localised prostate cancer, as well as the choice of AS for men with CPG2 disease. Additionally, they suggest AS as an option for men with CPG3 disease if they choose not to have immediate treatment [5]. There is broad international guideline endorsement, including recommendations from the European Association of Urology (EAU) to offer AS as the standard of care for low-risk disease [6] and the American National Comprehensive Cancer Network (NCCN), highlighting AS as the preferred option for men with very-low-risk prostate cancer and for most men with low-risk prostate cancer [7]. Nevertheless substantial variation remains in how patients are selected and how AS is implemented across real-world clinical settings [8]. The Movember Prostate Cancer Landscape Analysis emphasised the need for improved tools to support patient selection, risk stratification, and surveillance protocols [9].

Scientific and technological advancements in prostate cancer diagnostics [10], the emergence of personalised follow-up plans [11,12], healthcare system-based prioritisation of remote outpatient follow-up appointments [13], and the increased adoption of digital health records [14] have all contributed to the evolving landscape of AS. However, despite improvements in imaging and biopsy techniques, misclassification at diagnosis and limitations in terms of the ability to predict true disease progression during follow-up remain significant challenges in contemporary AS practice.

In this narrative review, we critically appraise recent evidence of molecular and genetic biomarkers in AS, focusing on their potential to refine patient selection and to predict disease progression during surveillance. We searched PubMed and Embase for English-language peer-reviewed studies published between 2000 and 2025, supplemented by international guidelines (EAU, AUA, NCCN, NICE) and a manual review of reference lists. Priority was given to large prospective cohorts, meta-analyses, and high-impact journals.

## 2. Context for Biomarkers: Long-Term Oncological Outcomes of AS

AS demonstrates excellent long-term oncological outcomes in men with low and favourable intermediate-risk prostate cancer [15,16,17,18,19,20,21,22,23,24,25,26,27,28]. In the latest update from the Canary Prostate Active Surveillance Study (*n* = 2155; median follow-up 7.2 years), 49% received treatment at 10 years, yet prostate cancer-specific mortality was only 0.1% [26]. Similar results have been reported across other large AS cohorts, including PRIAS (*n* = 5302) [18], the Toronto cohort (*n* = 993) [17], and the MSKCC cohort (*n* = 2664) [21], which all report 10-year prostate cancer-specific survival rates exceeding 98% (Supplementary Materials: Table S1).

Data from the Movember Foundation’s Global Action Plan (GAP3) consortium, encompassing 25 cohorts across 15 countries, support the long-term safety of AS for low- and intermediate-risk prostate cancer [29,30]. Among 14,623 men, 91% had Gleason 3 + 3 disease, 8% had Gleason 3 + 4 disease, and 1% had Gleason 4 + 3 disease. At 10 years, treatment had been initiated in 20% of men with low-risk and 31% with intermediate-risk disease. Most deaths were unrelated to prostate cancer, with 10-year overall survival at 84.1% and metastasis-free survival at 99.4% [30]. Predictors of grade reclassification at re-biopsy were age, PSA, prostate volume, T-stage, and number of positive biopsy cores [31]. Rates of AS discontinuation due to progression at 5 years were similar in men ≤ 60 and >60 years (22% vs. 25%) [32]. A more recent analysis of over 24,000 patients in the GAP3 AS cohort demonstrated a 15-year prostate cancer-specific survival of 98.7% and an overall survival of 88.5% [27].

In a UK single-centre prospective AS cohort from the Royal Marsden (*n* = 471), the 5-year adverse histology rate was 22% and treatment-free probability was 70% [28]. The ProtecT trial was a major UK-based multicentre RCT that, whilst not incorporating protocolised re-biopsy, provided further reassurance regarding conservative management. At 15 years, prostate cancer mortality was 3.1% in the active monitoring arm (*n* = 545), not significantly different from radical prostatectomy (2.2%) or radiotherapy (2.9%) (*p* = 0.53). Notably, 24.4% of men remained treatment-free at 15 years [23].

Long-term data have also been reported from the Cancer of the Prostate Strategic Urologic Research Endeavour (CaPSURE) registry [24]. In this prospective, multi-centre cohort (median follow-up 9.4 years), after adjustment for Cancer of the Prostate Risk Assessment (CAPRA) score, the hazard ratio for prostate cancer specific mortality (with radical prostatectomy as the reference) was 1.76 [95% confidence interval (CI): 1.30–2.40; *p* < 0.001] for the “monitoring” group, which included patients either on AS or under watchful waiting [24].

Although long-term survival outcomes are excellent, a substantial proportion of men transition to active treatment over time. Across cohorts, the 5-year conversion to treatment rate ranges from 24% to 52% (Supplementary Materials: Table S1), with histological upgrading now the most common trigger for intervention in the USA [7]. Earlier AS cohorts often relied on PSA kinetics to monitor disease, often without MRI imaging or re-biopsy, likely underestimating the true incidence of pathological progression. In addition, most long-term oncological outcome data is predominantly derived from white cohorts, limiting generalisability to more diverse populations. GAP3 analyses indicate higher risks of upgrading and progression in men of African ancestry, and suggest that rates of treatment vary by ethnicity independent of progression [33]. Together, these limitations highlight persistent challenges in the diagnosis, monitoring, and consistency of practice, providing the rationale for evaluating molecular biomarkers as complementary tools in AS.

## 3. Current Challenges in AS: Opportunities for Biomarkers

### 3.1. Patient Selection and Uptake of AS: The Misclassification Problem

Risk assessment tools are central to identifying suitable candidates for AS (i.e., selecting men with indolent disease that is unlikely to progress) and guiding long-term monitoring strategies. The modern diagnostic pathway entails pre-biopsy MRI followed by targeted prostate biopsy of suspicious lesions. Major international urological guidelines stratify newly diagnosed patients based on PSA, clinical stage, Gleason scoring, and biopsy factors, with tiered management recommendations (Table 1) [5,6,7,34]. The three-tiered risk classification system originally described by D’Amico, upon which the EAU and NCCN classifiers were derived from, predicted biochemical recurrence amongst men undergoing radical treatment for clinically localised prostate cancer [35]. In contrast, the Cambridge Prognostic Group (CPG) five-tiered model was built to predict cancer-specific mortality and derived from a prostate cancer cohort that included men managed with AS [36]. Importantly, CPG2 disease is associated with a better prognostic outlook than CPG3 disease. As evidenced by the NPCA group, the incorporation of the CPG model into UK practice has broadened eligibility for AS [37]. The UK currently has one of the highest rates of AS uptake worldwide, with only 8% of men diagnosed with low-risk disease in England undergoing radical treatment within 12 months of diagnosis [38]. This is in contrast to just under 60% in North America [39].

Differences in AS uptake globally reflect, in part, variation in classification systems and prognostic tools. The PREDICT Prostate model, used in the UK, advances traditional risk classification by incorporating patient age and co-morbidity status, in addition to standard diagnostic investigations, into a multivariable prognostic model. It estimates prostate cancer-specific and overall survival for patients undergoing conservative versus radical treatment, aiding decision-making for patients considering AS [40,41]. The PREDICT Prostate online tool can assist patient counselling by providing visual representations of survival as well as treatment harms (erectile dysfunction, incontinence, bowel issues) when comparing conservative versus radical treatment. The use of PREDICT resulted in a reduced chance of undergoing radical prostate surgery in patients with CPG2 disease [42]. In an RCT of standard information versus the structured presentation of the PREDICT prostate tool, PREDICT reduced patient decisional conflict and uncertainty when deciding about treatment [43].

Despite advances in imaging and biopsy techniques, misclassification at diagnosis remains a major challenge. Recent meta-analysis showed that the pooled rate of upgrading at the first confirmatory biopsy (performed between 4 and 24 months from diagnosis) among men AS fulfilling low- and intermediate-risk criteria is 20% [44]. In a cohort of men with CPG1–3 disease, confirmatory transperineal template biopsy resulted in up-classification or Gleason GG increase in 37.5% of patients (with 12.5% reclassified to ≥GG3 and 5% reclassified to ≥GG4) [45]. Furthermore, whilst pre-biopsy mpMRI has improved detection of clinically significant disease [46,47], there is concern that MRI-targeted biopsies may result in grade inflation and therefore reduce the number of men going into AS due to a higher perceived risk of disease progression [48]. The misclassification problem represents a key opportunity for biomarkers, which may help to identify multifocal aggressive disease at baseline and complement existing clinical tools.

### 3.2. Predicting Disease Progression During AS

Several modelling tools (Table 2) have been developed to predict likelihood of disease progression during AS. The PRIAS model, predicting cancer progression to ≥GG2, was based on data from the PRIAS study (patients with GG1 disease who were diagnosed using systematic biopsies in the absence of MRI) [49]. The Canary model was developed using the Canary PASS cohort (patients with GG1 disease) and incorporates PSA, biopsy results, time since diagnosis, BMI, and prostate size to predict a risk of ≥GG2 [50]. The Johns Hopkins model was devised based upon their very-low-risk cohort (Epstein criteria), which predicts the Gleason grade after radical prostatectomy [51]. Other clinical parameters have shown prognostic value. The STRATCANS model incorporates PSA density and the Cambridge Prognostic Group (CPG) at the start of AS to predict disease progression from CPG1-2 to CPG3 disease [52,53,54]. The 5-year STRATCANS outcomes were reported recently for 297 men with CPG1 or 2 disease on AS; treatment rates for CPG ≥ 3 disease were 4.7%, 12.9% and 27.4% for STRATCANS tiers 1, 2, and 3, respectively (*p*  <  0.001), with an area under the curve (AUC) of 0.74 for predicting CPG ≥ 3 progression [54].

In summary, performance across the available clinical models is similar and there is room for improvement, with AUCs ranging from 0.6 to 0.74 [50,51,55,56]. Interestingly, the US-based studies all describe progression to GG2 as an endpoint, whereas many patients with GG2 will still be suitable for AS. The consequence is that some men progress in the absence of clear risk factors, while others may undergo unnecessary intervention. Incorporation of molecular biomarkers may provide complementary biological information beyond clinical and imaging variables, potentially improving model performance and enabling the more individualised prediction of disease progression.

**Table 2:** Summary of existing clinical models for predicting progression in AS.

Model/Tool	Inclusion Criteria for Model Development Cohort	Pre-Biopsy MRI in Model Development Cohort	Disease Progression Endpoint	Model Performance
Canary model [57]	GG1	Not specified	Time from confirmatory biopsy to reclassification (GG2 or higher on subsequent biopsy)	AUC 0.70 (95% CI: 0.63–0.76)
John Hopkins [51]	Epstein criteria for very-low-risk prostate cancer	No	Radical prostatectomy pathological Gleason Score > 6	AUC 0.74 (95% CI 0.66–0.81)
PRIAS [55]	GG1 (on systematic biopsy)	No	Upgrading to GG2 or higher on repeat biopsy	AUC 0.6–0.7 (95% CI not reported)
STRATCANS [56]	CPG 1–2	Most (16% did not have MRI)	Progression to ≥CPG3	AUC 0.74 (95% CI: 0.63–0.85) In 5-year follow-up cohort (*n* = 297) c-index 0.724 (95% CI 0.694–0.793) on internal validation cohort (*n* = 883) c-index 0.845 (95%CI 0.712–0.958) on external validation cohort (*n* = 151)

Abbreviations: MRI, magnetic resonance imaging; GG, Gleason Grade Group; PRIAS, Prostate Cancer Research International Active Surveillance study; CPG, Cambridge Prognostic Group; AUC, area under the curve; CI, confidence interval; c-index, concordance index.

### 3.3. Heterogeneity in AS Clinical Practice

AS clinical practice is highly heterogeneous, complicating both the interpretation of the literature and the implementation of any innovations. There is variation across the full spectrum of AS delivery, from patient entry to monitoring protocols through to exit criteria and triggers for treatment.

For entry criteria, the GAP3 consortium has shown wide variation internationally in who is considered eligible for AS [30,58,59]. The recent Prostate Cancer UK AS policy report, based on freedom of information (FOI) requests, highlighted frequent deviation from NICE NG131 guidelines, with some centres incorporating PSA density or a number of positive biopsy cores in addition to the CPG group [8]. Consensus efforts such as the Movember International Consensus Meeting, building on the 2019 DETECTIVE statements, convened a multidisciplinary panel of healthcare professionals and 12 participants with lived experience of AS to define best practices [60,61]. Gleason grade and MRI findings were deemed the most important AS selection criteria, followed by PSA density and PSA. However, these conclusions remain opinion-based, and variation persists.

In terms of monitoring intensity, surveillance protocols are inconsistent. More frequent biopsy schedules during AS can deter some men from continuing AS despite not displaying grade progression [58]; data from the GAP3 consortium suggests that 12.8% of men convert to active treatment without evidence of disease progression [59]. Whilst the NICE guidance suggests a one-size-fits-all time-based schema of follow-up, in reality, many UK centres are now using stratified follow-up (only 39% of centres in the PCUK AS policy report were using a uniform follow-up protocol for all AS patients) [8]. Indeed, there is an increasing body of evidence to support the adoption of personalised follow-up schedules to enable men with the lowest risk disease to undergo a less intensive AS follow-up schedule [53,62,63].

In the UK, the STRATCANS model stratified men with CPG1–2 disease into 3 tiers with increasing follow-up intensity, and resource modelling suggests this could reduce clinic appointments by 22% and MRI scans by 42% compared to NICE guidelines [53]. This approach has now been incorporated into the 2025 Irish National Cancer Control Programme guidelines [64]. Englman et al. described an MRI-led risk-adapted protocol based on a retrospective cohort of 1150 patients on AS [62]. In their study, progression was defined as histological upgrading to GS ≥ 4 + 3 or transition to treatment. MRI was performed at baseline and 12 months, with an additional 24-month scan for men with a visible lesion at diagnosis. Repeat biopsy was reserved for those with radiological progression or rising PSA density, illustrating a shift towards imaging-led, risk-adapted AS. Future approaches to risk-stratified AS may incorporate dynamic surveillance rules, whereby sequential surveillance decisions rules adapt over time according to a patient’s evolving characteristics [63]. The increasing adoption of digital health records will provide the infrastructure to facilitate this.

Despite these efforts, there are inherent limits to how far clinical guidelines, consensus statements, or even validated diagnostic tools can standardise AS practice. Ultimately, decisions about entry, monitoring intensity, and the timing of intervention are shaped not only by clinician discretion but also by patient preference. This reliance on individual judgement and choice introduces inevitable variation, highlighting both the importance of robust clinical governance and the potential role of biomarkers as complementary tools to support, rather than replace, shared decision-making. Emerging biomarkers may help address these challenges by reducing misclassification, improving the prediction of progression, and offering greater objectivity within heterogeneous practice.

## 4. Improving Patient Selection to AS by Reducing Misclassification at Diagnosis

Appropriate patient selection to AS relies on accurate cancer classification at diagnosis. Systematic biopsy can be subject to the under-sampling of multifocal cancer leading to misclassification, which may later be mistaken for disease progression in AS [65]. As many as 36% of cases may be undergraded from biopsy pathology [66]. Over the past decade, several molecular and genomic biomarkers have been evaluated to improve diagnosis and refine AS eligibility. Biomarkers with evidence published in the last 10 years are summarised in Table 3.

### 4.1. Blood Biomarkers

The Prostate Health Index (PHI) combines total PSA, free PSA, and [−2] proPSA [71,85]. While recent studies supported its ability to predict pathological upgrading upon serial biopsy during AS (AUC 0.68, 95% CI: 0.53–0.82) [68,69], analysis from the Canary PASS cohort suggested PHI did not have better discrimination than clinical data alone for predicting reclassification upon subsequent re-biopsy [70].

The 4-kallikrein (4K) test, which is another PSA-based combinatory marker (total PSA, free PSA, intact PSA and human kallikrein-related peptidase 2 [hK2]) [86], predicted tumour reclassification by upgrading at confirmatory biopsy at 6 months in a cohort of 137 patients on AS who had ISUP GG1 at diagnosis [67]. Using a 7.5% cut-off, there were no re-classifications to GG3 on confirmatory biopsy, and biopsy rates could theoretically be reduced by 27%. The 4K score has also been incorporated alongside MRI in the Finnish ProScreen trial, a population-based study, where 1 additional low-grade cancer was detected per 909 men randomised to screening [87].

The Stockholm3 test combines plasma protein markers (hK2, microseminoprotein beta [MSMB], microphage inhibitory cytokine-1 [MIC1], total PSA and free PSA) with 101 single-nucleotide polymorphisms as well as clinical data [88]. In 280 AS patients with GG1 disease from the STHLM3 study, Stockholm3 discriminated reclassification to ≥GG2: among men with a negative test, only 7.9% had GG2 disease and none had unfavourable intermediate risk [72]. More recently, in a smaller AS cohort, a Stockholm3 cut-off ≥ 15 achieved 87.5% sensitivity and 90% NPV for upgrading at confirmatory biopsy [89].

### 4.2. Urine Biomarkers

Biomarkers that predict upgrading at prostatectomy may help address cancer misclassification. Biopsy samples represent only a small proportion of the gland, whereas prostate cancer is frequently multifocal. Molecular biomarkers may capture signals arising from multiple tumour foci, potentially providing complementary biological information beyond what is obtained from individual biopsy cores. The ExoDx Prostate Intelliscore (EPI) detects urinary ERG and PCA3 RNA relative to SPDEF [90]. In men with GG1 on biopsy, lower EPI scores were associated with concordant prostatectomy histology, while higher scores predicted upgrading to ≥GG3 (*p* < 0.001) [73]. Notably, no man with GG1 and EPI < 15.6 upgraded to ≥GG3, suggesting reassurance in selecting AS.

Erdmann et al. described their urinary EV-based marker [AMACR, HPN, MALAT1, PCA3 and PCAT29 transcripts combined with PSA density and MRI Prostate Imaging Reporting and Data System (PI-RADS)] [91]. In 72 patients (93% GG1, 7% GG2), the model achieved an AUC of 0.869 (*p* < 0.001) for Gleason reclassification. However, patients entered at variable time points, up to 12 years after diagnosis, limiting application to the clinic [91] as well as making comparability across studies challenging.

### 4.3. Tissue Biomarkers

The Genomic Prostate Score (GPS, Oncotype Dx) is a 17 gene classifier [83,92]. In 131 men on AS who had GPS, each 5-unit increase in GPS was associated with increased risk of upgrading at repeat biopsy [Hazard Ratio (HR) 1.28, 95% CI: 1.19–1.39, *p* < 0.01] [81]. In Canary PASS, however, adding GPS to PSA density and biopsy grade did not improve the prediction of adverse pathology at prostatectomy [82], making routine clinical use harder to justify.

Prolaris is a 46-gene assay that generates a Cell Cycle Progression (CCP) score from 0 to 10, with higher scores indicating higher progression risk [93,94], and has been shown to assist risk stratification in the context of patients who were upgraded to Gleason 7 at prostatectomy [95]. ProMark, an 8-protein immunofluorescence assay, aims to predict Gleason ≥ 4  +  3 and non-organ-confined disease in patients with Gleason 3  +  3 and 3  +  4 on prostate biopsy [96]. In a study comparing biopsy with matched prostatectomy specimens, ProMark had an AUC of 0.65 [*p* < 0.0001; Odds Radio (OR), 12.95] for distinguishing Gleason 6 vs. non-Gleason 6 pathology [97].

Decipher, a 22 gene assay originally developed for post-prostatectomy prognostication, has also featured in multiple recent studies relevant to AS selection [98,99]. In 647 men with NCCN very-low/low-risk or favourable/intermediate-risk prostate cancer undergoing prostatectomy, Decipher independently predicted adverse pathology (≥GG3, ≥pT3b, or N1; OR 1.34 per 0.1-unit increase), even after CAPRA adjustment [57]. However, this was observational and did not inform AS entry decisions directly.

## 5. Improving the Prediction of True Disease Progression During AS

Beyond accurate classification at diagnosis, a central challenge in AS is predicting true biological disease progression. In Section 3.2, we considered clinical models for predicting disease progression during AS; here, we evaluate how novel biomarkers may add predictive value during AS.

### 5.1. Blood Biomarkers

PSA kinetics have limited predictive value for grade progression during AS [100,101,102,103]. PHI at diagnosis has shown some ability to predict progression (defined as >3 positive cores or a Gleason score > 6 on follow-up biopsy) among a cohort of patients with ≤3 cores of Gleason 6 disease at diagnosis, with an AUC of 0.641 (95% CI: 0.51–0.77, *p* = 0.034) [104]. In terms of serial PHI testing, in 241 GG1 patients on AS, PHI risk category changes over time predicted grade re-classification (risk category 4 vs. 1, HR 4.2, 95% CI: 1.76–10.05, *p* = 0.002, C-index 0.759) [71].

The 4K score was evaluated in 166 patients. Two definitions of disease progression were used: “protocol-defined progression” (>4 cores with any grade cancer, >2 cores with GG2, any core with ≥GG3, GG1 upgraded to GG2, or any treatment irrespective of histology), and “grade progression” (GG1 upgraded to ≥GG, or GG2 upgraded to ≥GG3). Overall, 83 men progressed per protocol; only 6% were GG2 at diagnosis. A very recent 4Kscore  ≥  20% significantly predicted protocol-defined progression (OR 2.61, 95% CI: 1.03–6.63, *p*  =  0.044) and grade progression (OR  =  5.13, 95% CI: 1.63–16.11, *p*  =  0.005) [105].

Circulating microRNAs, small non-coding RNAs implicated in the biology of prostate cancer progression, can be detected in plasma and serum [106]. The 3-miR score (miR-223, -24, -375) independently predicted progression (Gleason upgrade, PSA doubling time < 3 years or PIRADS 4/5 on MRI) [107]. When combined with PSA in a multivariable model, the AUC for predicting disease progression was 0.70 (95% CI: 0.682–0.884).

### 5.2. Urine Biomarkers

SelectMDx detects urinary DLX1 and HOXC6 RNA, in combination with serum PSA and clinical data [108]. In 86 men on AS with low- or very-low risk prostate cancer, SelectMDx predicted pathological progression-free survival (progression defined as an increase in ISUP GG, tumour volume increase [>3 positive cores and >3 prostatic areas involved] or any core with >5 mm or >50% involvement), independently of the diagnostic grade [76]. Using an optimal cut-off of 5, patients with a SelectMDx score > 5 had an HR of 3.30 (95% CI: 1.75–6.24) for pathological progression at 5 years.

Urinary PCA3 has not demonstrated utility for predicting progression. In a cohort restricted to low-risk criteria (cT1c, PSA density < 0.15, GS6, ≤2 cores, ≤50% involvement), PCA3 alone did not discriminate men with progression (AUC 0.589, 95% CI: 0.50–0.68, *p* = 0.076) [109].

The Prostate Urine Risk (PUR) test evaluates 36 extracellular vesicle (EV) RNA transcripts, generating four risk signatures, from PUR-1 to PUR-4, aligned with D’Amico risk groups [74]. The test was found to predict disease progression in AS [75]. Among men on AS, the proportion of PUR-4 was significantly associated with outcomes, and a cut-off of 0.174 dichotomised patients with a marked difference in time to progression (HR 8.2, 95% CI: 3.26–20.81) [74]. The potential for serial testing is of interest. In a pilot cohort (*n* = 20), men who progressed were more likely to fail the PUR stability test (*p* = 0.059), suggesting value for longitudinal monitoring. This is currently being investigated in a multicentre study using home collection kits [74].

### 5.3. Tissue Biomarkers

Building upon previous evidence suggesting that the CCP (Prolaris) score could influence urologist treatment decision-making [110], in a multicentre retrospective observational study of men with localised prostate cancer who underwent CCP testing to select AS, two thirds of patients remained on AS for more than 3 years [84].

Vince et al. evaluated 264 patients who underwent Decipher biopsy prior to AS [77]. After adjusting for the NCCN risk group, age, PSA, prostate volume, body mass index, and percent positive cores, a high-risk Decipher score was independently associated with shorter time to treatment (HR 2.51, 95% CI: 1.52–4.13, *p* < 0.001). Press et al. found Decipher predicted upgrading among patients with diagnostic GG1 disease, but not GG2 (AUC 0.69, 95% CI: 0.58–0.80) [78]. Zhu et al. analysed SEER (Surveillance, Epidemiology and End Results registry) data including 2576 Decipher-tested and 84,564 untested men with GG1–2 disease [79]. Higher Decipher scores were associated with upgrading (OR 1.29, 95% CI: 1.12–1.49, *p* < 0.001), upstaging (OR 1.31, 95% CI: 1.05–1.62, *p* = 0.020), and adverse pathology (OR 1.27, 95% CI: 1.12–1.45, *p* < 0.001) [79].

Beyond baseline testing, tissue-based assays from repeat biopsies may add information. In 111 GG1 men with two GPS results, both first and second scores were associated with upgrading (≥GG2) and conversion to treatment [83]. Serial Decipher testing has also been described in a case report, with a doubling in score corresponding to reclassification on a third biopsy [111].

While these studies suggest promise, translation into routine AS remains inconsistent. A qualitative study highlighted mixed patient experiences: some found genomic testing helpful, while others reported poor communication about purpose and meaning [80]. Reflecting this uncertainty, AUA guidelines advise against the routine use of tissue-based genomic classifiers for AS, though selective use may be considered (e.g., high-volume GG1 or favourable intermediate-risk patients) [77]. Cost-effectiveness remains a critical barrier, and none of these assays are endorsed by NICE for NHS use at the time of writing.

Patient preference is an important factor in decision-making. Embedding clear counselling about the safety of AS and expected triggers for treatment may mitigate avoidable early conversions to treatment in the absence of biological disease progression, while still respecting patient autonomy. Shared decision-making frameworks, supported by decision aids such as PREDICT Prostate, may help differentiate anxiety-driven decisions from clinically indicated progression [42].

Several caveats must be acknowledged. Studies differ in their clinical endpoints, with some using treatment initiation, which may itself be influenced by genomic results, confounding associations with treatment-free survival. Many cohorts describe very conservative low-risk populations (predominantly GG1/CPG1), limiting generalisability to broader AS practice. In addition, most US series predate routine MRI prior to biopsy, raising the possibility that some biomarkers are used to detect baseline misclassification rather than true progression.

In contemporary clinical practice, men entering AS typically follow a structured pathway comprising pre-biopsy MRI, targeted and systematic biopsy at diagnosis, and confirmatory biopsy at around 12 months, with subsequent surveillance incorporating regular PSA testing, interval MRI and repeat biopsy at predefined time points. As we have explored, within this care pathway, different biomarkers may have distinct roles. Firstly, biomarkers such as PHI, 4Kscore, ExoDx and Stockholm3 may be utilised at, or shortly after, diagnosis to address potential misclassification and to support decision-making around suitability for earlier confirmatory biopsy and the consideration of immediate radical treatment. Secondly, biomarkers may assist the personalisation of AS protocols. For example, patients with urinary markers, suggesting higher risk of disease progression (e.g., Select MDx > 5, or PUR-4 score > 0.174), may warrant a more intensive surveillance protocol with more frequent MRI and biopsy, or indeed lower-risk results may support de-escalation. Finally, serial biomarker testing may have the potential to complement, and in some settings reduce, reliance on the current PSA-MRI-biopsy paradigm of AS; however, prospective studies embedded within standardised AS pathways are required to determine when such tests add incremental value and how they should be integrated alongside established clinical tools.

## 6. Conclusions

Emerging evidence suggests that molecular and genetic biomarkers at diagnosis and serially during AS may provide additional prognostic information. However, current studies remain small, use surrogate endpoints such as biopsy upgrading, and have limited follow-up. Priorities for future research include identifying the clinically relevant optimal biomarker cut-offs, understanding how biomarkers and MRI results interact, and exploring how they can be integrated to improve disease characterisation and guide AS decisions. The attainment of consensus has been slowed by heterogeneity in study design and endpoint definitions, as well as by a lack of validation in modern AS cohorts.

The major hurdle for novel biomarkers is their ability to demonstrate not only predictive accuracy but also clinical utility and cost-effectiveness, as well as sufficient incremental benefit over established current clinical tools: reducing unnecessary biopsies without compromising oncological safety, providing value for healthcare systems, ensuring equitable access, and enhancing communication with patients. Importantly, genomic biomarker development may also enhance biological understanding and provide mechanistic insights of disease progression. The transition towards remote care offers an opportunity for home sampling, e.g., via postal urine kits, integrated with digital records to potentially deliver risk-adapted follow-up that is both sustainable and acceptable. If validated in modern cohorts, the future of AS may involve novel biomarker combinations that transform surveillance from a “one-size-fits-all” protocol into a biologically informed personalised approach that maintains safety while minimising harm, fulfilling the central aim of modern prostate cancer management.

## 7. Patents

C.S.C. and D.S.B. have filed patent applications related to the Prostate Urine Risk (PUR) test, DESNT, and an anaerobic bacteria biomarker set for prostate cancer diagnosis and prognosis.

## Figures and Tables

**Table 1:** Summary of American (AUA, NCCN), British (NICE) and European (EAU) guidelines for risk stratification and AS recommendations.

Guideline	Risk Group	Clinical Criteria	Guideline Recommendation on AS
AUA [34]	Low Risk	ISUP GG1 and PSA < 10 ng/mL and cT1–T2a	Recommend AS as the preferred management option
	Intermediate Risk, Favourable	GG1 and PSA 10–<20 ng/mL OR cT2b-c and <50% biopsy cores positive OR GG2 with PSA < 10 ng/mL and cT1-2a and <50% biopsy cores positive	Discuss AS and radical therapy
EAU [6]	Low Risk	ISUP GG1 and PSA < 10 ng/mL and cT1-2a	Recommended AS as standard of care
	Favourable Intermediate Risk	ISUP GG2 and PSA < 10 ng/mL and cT1-2b OR ISUP GG1 and PSA 10–20 ng/mL and cT1-2b OR ISUP GG1 and PSA < 10 ng/mL and cT2b	AS may be considered in selected cases
NCCN [7]	Very Low Risk	Has all of the following: cT1c ISUP GG1 PSA < 10 ng/mL <3 prostate biopsy fragments/cores positive, ≤50% cancer in each fragment/core PSA density < 0.15 ng/mL/g	Recommend AS as the preferred management option
	Low Risk	Has all of the following but does not qualify for very low risk: cT1–cT2a ISUP GG1 PSA < 10 ng/mL	Recommend AS as the preferred management option for most patients
	Favourable Intermediate Risk	Has all of the following: 1 risk intermediate risk factor (cT2b–cT2c, GG2 or 3, PSA 10–20 ng/mL) ISUP GG1 or 2 <50% biopsy cores positive (e.g., <6 of 12 cores)	Offer AS to carefully selected patients
NICE [5]	Cambridge Prognostic Group 1	ISUP GG1 and PSA < 10 ng/mL and stages cT1–T2	Offer AS Consider radical treatment if AS not suitable or acceptable to the person
	Cambridge Prognostic Group 2	ISUP GG2 OR PSA 10–20 ng/mL and stages cT1–T2	Offer a choice between AS or radical treatment if radical treatment is suitable
	Cambridge Prognostic Group 3	ISUP GG2 and PSA 10–20 ng/mL and stages cT1–T2 OR ISUP GG3 and stages cT1–T2	Offer radical treatment and consider AS for people who choose not to have immediate radical treatment

Abbreviations: AS, active surveillance; AUA, American Urological Association; NCCN, National Comprehensive Cancer Network; NICE, National Institute for Health and Care Excellence; EAU, European Association of Urology; ISUP, International Society of Urological Pathology; GG, Gleason Grade Group; PSA, prostate-specific antigen; cT, clinical T-stage (TNM classification).

**Table 3:** Summary of biomarkers that are relevant to AS selection and monitoring with evidence published in the last 10 years. The cited studies are described in detail, with clinical endpoints, in Section 3 and Section 4.

Name of Biomarker	Sample Type	Description of Biomarker +/− Clinical Factors Incorporated into Algorithm	Technical Notes/Methodology	Readout	FDA Approval Status	Commercial Availability	Roles in AS with Recent Evidence
4-kallikrein (4K)	Blood	Total PSA, Free PSA, Intact PSA, human kallikrein-related peptidase 2 (hK2), combined with clinical factors (prior biopsy status, age and DRE)	Immunoassay	4Kscore from <1% to >95%	Approved	Yes	Patient selection [67]
Prostate Health Index (PHI)	Blood	Total PSA, Free PSA, [−2] proPSA	Immunoassay	Phi score calculated from the formula: ([−2]proPSA/free PSA) × √PSA	Approved	Yes	Patient selection [68,69,70] Serial monitoring [71]
Stockholm3	Blood	Plasma protein markers (human glandular kallikrein 2 [hK2], microseminoprotein beta [MSMB], microphage inhibitory cytokine-1 [MIC1], total PSA and free PSA) are combined with genetic markers (101 single-nucleotide polymorphisms) and clinical data (age, first-degree family history of prostate cancer, a previous biopsy, digital rectal examination, and prostate volume assessed by transrectal ultrasound at cancer diagnosis)	Immunoassay and RT-PCR	Stockholm3 score from 0% to 100%	Not approved by FDA (offered as laboratory-based test in a CLIA certified laboratory)	Yes	Patient selection [72]
ExoDx Prostate Intelliscore (EPI)	Urine (non-DRE)	EV ERG and PCA3 mRNA relative to SPDEF expression	RT-PCR	ExoDx Prostate Test score from 0 to 100	Approved	Yes	Patient selection [73]
Prostate Urine Risk (PUR)	Urine (post-DRE)	36-gene signature (AMACR, MEX3A, AMH, MEMO1, ANKRD34B, MME, APOC1, MMP11AR(exons 4–8), MMP26, DPP4, NKAIN1, ERG(exons 4–5), PALM3, GABARAPL2, PCA3, GAPDH, PPFIA2, GDF15, SIM2 (short), HOXC6, SMIM1, HPN, SSPO, IGFBP3, SULT1A1, IMPDH2, TDRD1, ITGBL1, TMPRSS2/ERG fusion, KLK4, TRPM4, MARCH5, TWIST1, MED4, UPK2)	NanoString expression analysis	Primary PUR score (PUR-1, PUR-2, PUR-3, PUR-4); PUR-4 as a continuous variable from 0 to 1	Not approved (Offered only in a research setting)	Not available outside of research setting	Patient selection [74,75] Serial monitoring [74]
SelectMDx	Urine (post-DRE)	DLX1 and HOXC6 mRNA, in combination with serum PSA and clinical factors (age, DRE, prostate volume, family history of prostate cancer)	RT-PCR	SelectMDx risk score from −6 to 6	Not approved by FDA (Offered as laboratory-based test in a CLIA certified laboratory)	Yes	Patient selection [76]
Decipher Prostate (22-gene genomic classifier, GC)	Biopsy tissue (FFPE)	22-gene signature (LASP1, IQGAP3, NFIB, S1PR4, THBS2, ANO7, PCDH7, MYBPC1, EPPK1, TSBP, PBX1, NUSAP1, ZWILCH, UBE2C, CAMK2N1, RABGAP1, PCAT-32, GLYATL1P4/PCAT-80, TNFRSF19)	Microarray	Genomic Classifier score from 0–1 (also known as the Decipher Biopsy score)	Not approved by FDA (Offered as laboratory-based test in a CLIA certified laboratory)	Yes	Patient selection [57,77,78,79,80]
Genomic Prostate Score (GPS)—previously Oncotype Dx	Biopsy tissue (FFPE)	17-gene signature (AZGP1, KLK2, SRD5A2, FAM13C, FLNC, GSN, TPM2, GSTM2, TPX2, BGN, COL1A1, SFRP4, ARF1, ATP5E, CLTC, GPS1 and PGK1)	RT-PCR	GPS from 0 to 100	Not approved by FDA (Offered as laboratory-based test in a CLIA certified laboratory)	Yes	Patient selection [81,82] Serial monitoring [83]
Prolaris biopsy test (Cell Cycle Progression score)	Biopsy tissue (FFPE)	46-gene signature (FOXM1, CDC20, CDKN3, CDC2, KIF11, KIAA0101, NUSAP1, CENPF, ASPM, BUB1B, RRM2, DLGAP5, BIRC5, KIF20A, PLK1, TOP2A, TK1, PBK, ASF1B, C18orf24, RAD54L, PTTG1, CDCA3, MCM10, PRC1, DTL, CEP55, RAD51, CENPM, CDCA8, ORC6L, and 15 housekeeping genes)	RT-PCR	Cell Cycle Progression score from 0 to 6	Approved	Yes	Patient selection [84]

Abbreviations: AS, active surveillance; PSA, prostate-specific antigen; DRE, digital rectal examination; EV, extracellular vesicle; ERG, ETS-related gene; PCA3, Prostate Cancer Antigen 3; mRNA, messenger ribonucleic acid; RT-PCR, reverse transcription polymerase chain reaction; FFPE, formalin-fixed paraffin-embedded; GC, genomic classifier; FDA, U.S. Food and Drug Administration; CLIA, Clinical Laboratory Improvement Amendments; NPV, negative predictive value.

## Data Availability

No new data were created or analysed in this study. Data sharing is not applicable to this article.

## Supplementary Materials

The following supporting information can be downloaded at: https://www.mdpi.com/article/10.3390/genes17010071/s1, Table S1: Summary of active surveillance cohorts with long-term survival data. References [15,16,17,18,19,20,21,22,23,24,25,26,27,28] are cited in the Supplementary Materials.

## Abbreviations

The following abbreviations are used in this manuscript:

AS	Active surveillance
AUA	American Urological Association
AUC	Area under the curve
BMI	Body mass index
CaPSURE	Cancer of the Prostate Strategic Urologic Research Endeavour
CAPRA	Cancer of the Prostate Risk Assessment
CCP	Cell Cycle Progression score
CI	Confidence interval
CPG	Cambridge Prognostic Group
DNA	Deoxyribonucleic acid
EAU	European Association of Urology
EPI	ExoDx Prostate Intelliscore
EV	Extracellular vesicle
FOI	Freedom of information
GAP3	Global Action Plan 3 (Movember Foundation consortium)
GG	Gleason Grade group
GPS	Genomic Prostate Score
GS	Gleason Score
hK2	Human kallikrein-related peptidase 2
HR	Hazard ratio
IQR	Interquartile range
ISUP	International Society of Urological Pathology
MIC1	Macrophage inhibitory cytokine-1
mpMRI	Multiparametric magnetic resonance imaging
MSMB	Microseminoprotein beta
NCCN	National Comprehensive Cancer Network
NICE	National Institute for Health and Care Excellence
NPCA	National Prostate Cancer Audit
NPV	Negative predictive value
OR	Odds ratio
PASS	Prostate Active Surveillance Study
PCA3	Prostate Cancer Antigen 3
PCUK	Prostate Cancer UK
PHI	Prostate Health Index
PI-RADS	Prostate Imaging Reporting and Data System
PRIAS	Prostate Cancer Research International Active Surveillance study
PSA	Prostate-specific antigen
PUR	Prostate Urine Risk
RCT	Randomised controlled trial
RNA	Ribonucleic acid
SD	Standard deviation
SEER	Surveillance, Epidemiology and End Results
STHLM3	Stockholm3
STRATCANS	Stratified Cancer Surveillance
T-stage	Tumour stage

## References

[1] Lardas M., Liew M., van den Bergh R.C., De Santis M., Bellmunt J., Van den Broeck T., Cornford P., Cumberbatch M.G., Fossati N., Gross T. (2017). Quality of Life Outcomes after Primary Treatment for Clinically Localised Prostate Cancer: A Systematic Review. Eur. Urol..

[2] Johansson E., Steineck G., Holmberg L., Johansson J.E., Nyberg T., Ruutu M., Bill-Axelson A. (2011). Long-term quality-of-life outcomes after radical prostatectomy or watchful waiting: The Scandinavian Prostate Cancer Group-4 randomised trial. Lancet Oncol..

[3] James Lind Alliance: Priority Setting Partnerships (2010). Prostate Cancer Top 10 Priorities.

[4] Bruinsma S.M., Roobol M.J., Carroll P.R., Klotz L., Pickles T., Moore C.M., Gnanapragasam V.J., Villers A., Rannikko A., Valdagni R. (2017). Semantics in active surveillance for men with localized prostate cancer—Results of a modified Delphi consensus procedure. Nat. Rev. Urol..

[5] National Institute for Health and Care Excellence (2019). Prostate Cancer Diagnosis and Management.

[6] EAU Guidelines on Prostate Cancer. Presented at the EAU Annual Congress, Madrid, Spain, 21–24 March 2025. https://uroweb.org/guidelines/urological-infections/chapter/citation-information.

[7] National Comprehensive Cancer Network (2025). NCCN Clinical Practice Guidelines in Oncology (NCCN Guidelines^®^) for Prostate Cancer.

[8] Naranjo A.E.L., Blake E., Hewett-Abbott G., Seggie A. (2025). Prostate Cancer Active Surveillance: Results and Policy Recommendations Following a UK Freedom of Information (FOI) Request. https://prostatecanceruk.org/media/plwi2fdx/active-surveillance-foi-policy-report_august_2025.pdf.

[9] Kouspou M.M., Fong J.E., Brew N., Hsiao S.T.F., Davidson S.L., Choyke P.L., Crispino T., Jain S., Jenster G.W., Knudsen B.S. (2020). The Movember Prostate Cancer Landscape Analysis: An assessment of unmet research needs. Nat. Rev. Urol..

[10] Vakili S., Beheshti I., Barzegar Behrooz A., Łos M.J., Vitorino R., Ghavami S. (2025). Transforming Prostate Cancer Care: Innovations in Diagnosis, Treatment, and Future Directions. Int. J. Mol. Sci..

[11] NHS England Personalised Follow-Up.

[12] Getting It Right First Time (GIRFT) (2022). Urology Outpatient Transformation: A Practical Guide to Delivery.

[13] NHS England (2022). Outpatient Recovery and Transformation Programme.

[14] NHS England (2025). Digital Transformation.

[15] Dall’Era M.A., Konety B.R., Cowan J.E., Shinohara K., Stauf F., Cooperberg M.R., Meng M.V., Kane C.J., Perez N., Master V.A. (2008). Active surveillance for the management of prostate cancer in a contemporary cohort. Cancer.

[16] Welty C.J., Cowan J.E., Nguyen H., Shinohara K., Perez N., Greene K.L., Chan J.M., Meng M.V., Simko J.P., Cooperberg M.R. (2015). Extended followup and risk factors for disease reclassification in a large active surveillance cohort for localized prostate cancer. J. Urol..

[17] Klotz L., Vesprini D., Sethukavalan P., Jethava V., Zhang L., Jain S., Yamamoto T., Mamedov A., Loblaw A. (2015). Long-term follow-up of a large active surveillance cohort of patients with prostate cancer. J. Clin. Oncol..

[18] Bokhorst L.P., Valdagni R., Rannikko A., Kakehi Y., Pickles T., Bangma C.H., Roobol M.J. (2016). A Decade of Active Surveillance in the PRIAS Study: An Update and Evaluation of the Criteria Used to Recommend a Switch to Active Treatment. Eur. Urol..

[19] Godtman R.A., Holmberg E., Khatami A., Pihl C.G., Stranne J., Hugosson J. (2016). Long-term Results of Active Surveillance in the Göteborg Randomized, Population-based Prostate Cancer Screening Trial. Eur. Urol..

[20] Tosoian J.J., Mamawala M., Epstein J.I., Landis P., Macura K.J., Simopoulos D.N., Carter H.B., Gorin M.A. (2020). Active Surveillance of Grade Group 1 Prostate Cancer: Long-term Outcomes from a Large Prospective Cohort. Eur. Urol..

[21] Carlsson S., Benfante N., Alvim R., Sjoberg D.D., Vickers A., Reuter V.E., Fine S.W., Vargas H.A., Wiseman M., Mamoor M. (2020). Long-Term Outcomes of Active Surveillance for Prostate Cancer: The Memorial Sloan Kettering Cancer Center Experience. J. Urol..

[22] Cooley L.F., Emeka A.A., Meyers T.J., Cooper P.R., Lin D.W., Finelli A., Eastham J.A., Logothetis C.J., Marks L.S., Vesprini D. (2021). Factors Associated with Time to Conversion from Active Surveillance to Treatment for Prostate Cancer in a Multi-Institutional Cohort. J. Urol..

[23] Hamdy F.C., Donovan J.L., Lane J.A., Metcalfe C., Davis M., Turner E.L., Martin R.M., Young G.J., Walsh E.I., Bryant R.J. (2023). Fifteen-Year Outcomes after Monitoring, Surgery, or Radiotherapy for Prostate Cancer. N. Engl. J. Med..

[24] Herlemann A., Cowan J.E., Washington S.L., Wong A.C., Broering J.M., Carroll P.R., Cooperberg M.R. (2024). Long-term Prostate Cancer-specific Mortality After Prostatectomy, Brachytherapy, External Beam Radiation Therapy, Hormonal Therapy, or Monitoring for Localized Prostate Cancer. Eur. Urol..

[25] Leclercq L., Bastide C., Lechevallier E., Walz J., Charvet A.L., Gondran-Tellier B., Campagna J., Savoie P.H., Long-Depaquit T., Daniel L. (2024). Active surveillance of low-grade prostate cancer using the SurACaP Criteria: A multi-institutional series with a median follow-up of 10years. Fr. J. Urol..

[26] Newcomb L.F., Schenk J.M., Zheng Y., Liu M., Zhu K., Brooks J.D., Carroll P.R., Dash A., de la Calle C.M., Ellis W.J. (2024). Long-Term Outcomes in Patients Using Protocol-Directed Active Surveillance for Prostate Cancer. JAMA.

[27] Tohi Y., Sahrmann J.M., Arbet J., Kato T., Lee L.S., Peacock M., Ginsburg K., Pavlovich C., Carroll P., Bangma C.H. (2025). De-escalation of Monitoring in Active Surveillance for Prostate Cancer: Results from the GAP3 Consortium. Eur. Urol. Oncol..

[28] Selvadurai E.D., Singhera M., Thomas K., Mohammed K., Woode-Amissah R., Horwich A., Huddart R.A., Dearnaley D.P., Parker C.C. (2013). Medium-term outcomes of active surveillance for localised prostate cancer. Eur. Urol..

[29] Bruinsma S.M., Zhang L., Roobol M.J., Bangma C.H., Steyerberg E.W., Nieboer D., Van Hemelrijck M. (2018). The Movember Foundation’s GAP3 cohort: A profile of the largest global prostate cancer active surveillance database to date. BJU Int..

[30] Bangma C., Doan P., Zhu L., Remmers S., Nieboer D., Helleman J., Roobol M.J., Sugimoto M., Chung B.H., Lee L.S. (2024). Has Active Surveillance for Prostate Cancer Become Safer? Lessons Learned from a Global Clinical Registry. Eur. Urol. Oncol..

[31] Bruinsma S.M., Nieboer D., Roobol M.J., Bangma C.H., Verbeek J.F.M., Gnanapragasam V., Van Hemelrijck M., Frydenberg M., Lee L.S., Valdagni R. (2021). Risk-Based Selection for Active Surveillance: Results of the Movember Foundation’s Global Action Plan Prostate Cancer Active Surveillance (GAP3) Initiative. J. Urol..

[32] Remmers S., Helleman J., Nieboer D., Trock B., Hyndman M.E., Moore C.M., Gnanapragasam V., Shiong Lee L., Elhage O., Klotz L. (2022). Active Surveillance for Men Younger than 60 Years or with Intermediate-risk Localized Prostate Cancer. Descriptive Analyses of Clinical Practice in the Movember GAP3 Initiative. Eur. Urol. Open Sci..

[33] Beckmann K., Santaolalla A., Helleman J., Carroll P., Ha Chung B., Shiong Lee L., Perry A., Rubio-Briones J., Sugimoto M., Trock B. (2021). Comparison of Characteristics, Follow-up and Outcomes of Active Surveillance for Prostate Cancer According to Ethnicity in the GAP3 Global Consortium Database. Eur. Urol. Open Sci..

[34] Eastham J.A., Auffenberg G.B., Barocas D.A., Chou R., Crispino T., Davis J.W., Eggener S., Horwitz E.M., Kane C.J., Kirkby E. (2022). Clinically localized prostate cancer: AUA/ASTRO guideline, part I: Introduction, risk assessment, staging, and risk-based management. J. Urol..

[35] D’Amico A.V., Whittington R., Malkowicz S.B., Schultz D., Blank K., Broderick G.A., Tomaszewski J.E., Renshaw A.A., Kaplan I., Beard C.J. (1998). Biochemical Outcome After Radical Prostatectomy, External Beam Radiation Therapy, or Interstitial Radiation Therapy for Clinically Localized Prostate Cancer. JAMA.

[36] Gnanapragasam V.J., Bratt O., Muir K., Lee L.S., Huang H.H., Stattin P., Lophatananon A. (2018). The Cambridge Prognostic Groups for improved prediction of disease mortality at diagnosis in primary non-metastatic prostate cancer: A validation study. BMC Med..

[37] Parry M.G., Cowling T.E., Sujenthiran A., Nossiter J., Berry B., Cathcart P., Aggarwal A., Payne H., van der Meulen J., Clarke N.W. (2020). Risk stratification for prostate cancer management: Value of the Cambridge Prognostic Group classification for assessing treatment allocation. BMC Med..

[38] NPCA *National Prostate Cancer Audit: State of the Nation Report*; NPCA: London, UK, 2025. https://www.npca.org.uk/wp-content/uploads/2025/01/NPCA-State-of-the-Nation-Report-2024_v2.pdf.

[39] Cooperberg M.R., Meeks W., Fang R., Gaylis F.D., Catalona W.J., Makarov D.V. (2023). Time Trends and Variation in the Use of Active Surveillance for Management of Low-risk Prostate Cancer in the US. JAMA Netw. Open.

[40] Thurtle D.R., Greenberg D.C., Lee L.S., Huang H.H., Pharoah P.D., Gnanapragasam V.J. (2019). Individual prognosis at diagnosis in nonmetastatic prostate cancer: Development and external validation of the PREDICT Prostate multivariable model. PLoS Med..

[41] Devos G., Joniau S. (2020). PREDICT Prostate, a useful tool in men with low- and intermediate-risk prostate cancer who are hesitant between conservative management and active treatment. BMC Med..

[42] Pandiaraja M., Pryle I., West L., Gardner L., Shallcross O., Tay J., Shah N., Gnanapragasam V., Lamb B.W. (2024). Utilisation and impact of predict prostate on decision-making among clinicians and patients in a specialist tertiary referral centre: A retrospective cohort study. BJUI Compass.

[43] Thurtle D., Jenkins V., Freeman A., Pearson M., Recchia G., Tamer P., Leonard K., Pharoah P., Aning J., Madaan S. (2021). Clinical Impact of the Predict Prostate Risk Communication Tool in Men Newly Diagnosed with Nonmetastatic Prostate Cancer: A Multicentre Randomised Controlled Trial. Eur. Urol..

[44] Mac Curtain B.M., Daly K., Calpin G., Collins E., Deshwal A., Lynch O., Qian W., O’Mahony A., Temperley H.C., Mac Curtain R.D. (2025). Reclassification of prostate cancer on first confirmatory prostate biopsy in men under active surveillance: A systematic review and meta-analysis. Cent. Eur. J. Urol..

[45] Gabb H., Gnanapragasam V.J. (2024). Value of a confirmatory re-biopsy as part of a modern risk stratified cancer surveillance programme for early prostate cancer. BJUI Compass.

[46] Kasivisvanathan V., Rannikko A.S., Borghi M., Panebianco V., Mynderse L.A., Vaarala M.H., Briganti A., Budäus L., Hellawell G., Hindley R.G. (2018). MRI-Targeted or Standard Biopsy for Prostate-Cancer Diagnosis. N. Engl. J. Med..

[47] van der Leest M., Cornel E., Israël B., Hendriks R., Padhani A.R., Hoogenboom M., Zamecnik P., Bakker D., Setiasti A.Y., Veltman J. (2019). Head-to-head Comparison of Transrectal Ultrasound-guided Prostate Biopsy Versus Multiparametric Prostate Resonance Imaging with Subsequent Magnetic Resonance-guided Biopsy in Biopsy-naïve Men with Elevated Prostate-specific Antigen: A Large Prospective Multicenter Clinical Study. Eur. Urol..

[48] Batouche A.O., Czeizler E., Lehto T.P., Erickson A., Shadbahr T., Laajala T.D., Pohjonen J., Vickers A.J., Mirtti T., Rannikko A.S. (2024). MRI-Targeted Prostate Biopsy Introduces Grade Inflation and Overtreatment. medRxiv.

[49] Tomer A., Nieboer D., Roobol M.J., Steyerberg E.W., Rizopoulos D. (2018). Personalized Schedules for Surveillance of Low-Risk Prostate Cancer Patients. Biometrics.

[50] Cooperberg M.R., Zheng Y., Faino A.V., Newcomb L.F., Zhu K., Cowan J.E., Brooks J.D., Dash A., Gleave M.E., Martin F. (2020). Tailoring Intensity of Active Surveillance for Low-Risk Prostate Cancer Based on Individualized Prediction of Risk Stability. JAMA Oncol..

[51] Coley R.Y., Zeger S.L., Mamawala M., Pienta K.J., Carter H.B. (2017). Prediction of the Pathologic Gleason Score to Inform a Personalized Management Program for Prostate Cancer. Eur. Urol..

[52] Gnanapragasam V.J., Barrett T., Thankapannair V., Thurtle D., Rubio-Briones J., Domínguez-Escrig J., Bratt O., Statin P., Muir K., Lophatananon A. (2019). Using prognosis to guide inclusion criteria, define standardised endpoints and stratify follow-up in active surveillance for prostate cancer. BJU Int..

[53] Thankapannair V., Keates A., Barrett T., Gnanapragasam V.J. (2023). Prospective Implementation and Early Outcomes of a Risk-stratified Prostate Cancer Active Surveillance Follow-up Protocol. Eur. Urol. Open Sci..

[54] Gnanapragasam V.J., Keates A., Lophatananon A., Thankapannair V. (2025). The 5-year results of the Stratified Cancer Active Surveillance programme for men with prostate cancer. BJU Int..

[55] Tomer A., Nieboer D., Roobol M.J., Bjartell A., Steyerberg E.W., Rizopoulos D. (2021). Personalised biopsy schedules based on risk of Gleason upgrading for patients with low-risk prostate cancer on active surveillance. BJU Int..

[56] Light A., Lophatananon A., Keates A., Thankappannair V., Barrett T., Dominguez-Escrig J., Rubio-Briones J., Benheddi T., Olivier J., Villers A. (2022). Development and External Validation of the STRATified CANcer Surveillance (STRATCANS) Multivariable Model for Predicting Progression in Men with Newly Diagnosed Prostate Cancer Starting Active Surveillance. J. Clin. Med..

[57] Herlemann A., Huang H.C., Alam R., Tosoian J.J., Kim H.L., Klein E.A., Simko J.P., Chan J.M., Lane B.R., Davis J.W. (2020). Decipher identifies men with otherwise clinically favorable-intermediate risk disease who may not be good candidates for active surveillance. Prostate Cancer Prostatic Dis..

[58] Beckmann K.R., Bangma C.H., Helleman J., Bjartell A., Carroll P.R., Morgan T., Nieboer D., Santaolalla A., Trock B.J., Valdagni R. (2022). Comparison of outcomes of different biopsy schedules among men on active surveillance for prostate cancer: An analysis of the G.A.P.3 global consortium database. Prostate.

[59] Van Hemelrijck M., Ji X., Helleman J., Roobol M.J., van der Linden W., Nieboer D., Bangma C.H., Frydenberg M., Rannikko A., Lee L.S. (2019). Reasons for Discontinuing Active Surveillance: Assessment of 21 Centres in 12 Countries in the Movember GAP3 Consortium. Eur. Urol..

[60] Moore C.M., King L.E., Withington J., Amin M.B., Andrews M., Briers E., Chen R.C., Chinegwundoh F.I., Cooperberg M.R., Crowe J. (2023). Best Current Practice and Research Priorities in Active Surveillance for Prostate Cancer—A Report of a Movember International Consensus Meeting. Eur. Urol. Oncol..

[61] Lam T.B.L., MacLennan S., Willemse P.M., Mason M.D., Plass K., Shepherd R., Baanders R., Bangma C.H., Bjartell A., Bossi A. (2019). EAU-EANM-ESTRO-ESUR-SIOG Prostate Cancer Guideline Panel Consensus Statements for Deferred Treatment with Curative Intent for Localised Prostate Cancer from an International Collaborative Study (DETECTIVE Study). Eur. Urol..

[62] Englman C., Adebusoye B., Maffei D., Stavrinides V., Bridge J., Kirkham A., Allen C., Dickinson L., Pendse D., Punwani S. (2025). Magnetic Resonance Imaging–led Risk-adapted Active Surveillance for Prostate Cancer: Updated Results from a Large Cohort Study. Eur. Urol..

[63] Dong X., Zheng Y., Lin D.W., Newcomb L., Zhao Y.Q. (2023). Constructing time-invariant dynamic surveillance rules for optimal monitoring schedules. Biometrics.

[64] National Cancer Control Programme HSE National Clinical Guideline: Active Surveillance for Patients with Prostate Cancer 2025. https://www2.healthservice.hse.ie/files/495/.

[65] Ahdoot M., Wilbur Andrew R., Reese Sarah E., Lebastchi Amir H., Mehralivand S., Gomella Patrick T., Bloom J., Gurram S., Siddiqui M., Pinsky P. (2020). MRI-Targeted, Systematic, and Combined Biopsy for Prostate Cancer Diagnosis. N. Engl. J. Med..

[66] Epstein J.I., Feng Z., Trock B.J., Pierorazio P.M. (2012). Upgrading and downgrading of prostate cancer from biopsy to radical prostatectomy: Incidence and predictive factors using the modified Gleason grading system and factoring in tertiary grades. Eur. Urol..

[67] Borque-Fernando Á., Rubio-Briones J., Esteban L.M., Dong Y., Calatrava A., Gómez-Ferrer Á., Gómez-Gómez E., Gil Fabra J.M., Rodríguez-García N., López González P. (2019). Role of the 4Kscore test as a predictor of reclassification in prostate cancer active surveillance. Prostate Cancer Prostatic Dis..

[68] Eng S.E., Basasie B., Lam A., John Semmes O., Troyer D.A., Clarke G.D., Sunnapwar A.G., Leach R.J., Johnson-Pais T.L., Sokoll L.J. (2024). Prospective comparison of restriction spectrum imaging and non-invasive biomarkers to predict upgrading on active surveillance prostate biopsy. Prostate Cancer Prostatic Dis..

[69] Schwen Z.R., Mamawala M., Tosoian J.J., Druskin S.C., Ross A.E., Sokoll L.J., Epstein J.I., Carter H.B., Gorin M.A., Pavlovich C.P. (2020). Prostate Health Index and multiparametric magnetic resonance imaging to predict prostate cancer grade reclassification in active surveillance. BJU Int..

[70] Filson C.P., Zhu K., Huang Y., Zheng Y., Newcomb L.F., Williams S., Brooks J.D., Carroll P.R., Dash A., Ellis W.J. (2022). Impact of Prostate Health Index Results for Prediction of Biopsy Grade Reclassification During Active Surveillance. J. Urol..

[71] de la Calle C.M., Jing Y., Mamawala M.M., Landis P., Macura K.J., Trock B.J., Epstein J.I., Sokoll L.J., Pavlovich C.P. (2023). Baseline prostate health index risk category and risk category changes during active surveillance predict grade reclassification. Urol. Oncol. Semin. Orig. Investig..

[72] Olsson H., Nordström T., Jäderling F., Egevad L., Vigneswaran H.T., Annerstedt M., Grönberg H., Eklund M., Lantz A. (2021). Incorporating Magnetic Resonance Imaging and Biomarkers in Active Surveillance Protocols-Results From the Prospective Stockholm3 Active Surveillance Trial (STHLM3AS). J. Natl. Cancer Inst..

[73] Kretschmer A., Tutrone R., Alter J., Berg E., Fischer C., Kumar S., Torkler P., Tadigotla V., Donovan M., Sant G. (2022). Pre-diagnosis urine exosomal RNA (ExoDx EPI score) is associated with post-prostatectomy pathology outcome. World J. Urol..

[74] Connell S.P., Yazbek-Hanna M., McCarthy F., Hurst R., Webb M., Curley H., Walker H., Mills R., Ball R.Y., Sanda M.G. (2019). A four-group urine risk classifier for predicting outcomes in patients with prostate cancer. BJU Int..

[75] (2025). Abstracts. Eur. Urol..

[76] Fiorella D., Marenco J.L., Mascarós J.M., Borque-Fernando Á., Esteban L.M., Calatrava A., Pastor B., López-Guerrero J.A., Rubio-Briones J. (2021). Role of PCA3 and SelectMDx in the optimization of active surveillance in prostate cancer. Actas Urol. Esp..

[77] Vince R.A., Jiang R., Qi J., Tosoian J.J., Takele R., Feng F.Y., Linsell S., Johnson A., Shetty S., Hurley P. (2022). Impact of Decipher Biopsy testing on clinical outcomes in localized prostate cancer in a prospective statewide collaborative. Prostate Cancer Prostatic Dis..

[78] Press B.H., Jones T., Olawoyin O., Lokeshwar S.D., Rahman S.N., Khajir G., Lin D.W., Cooperberg M.R., Loeb S., Darst B.F. (2022). Association Between a 22-feature Genomic Classifier and Biopsy Gleason Upgrade During Active Surveillance for Prostate Cancer. Eur. Urol. Open Sci..

[79] Zhu A., Proudfoot J.A., Davicioni E., Ross A.E., Petkov V.I., Bonds S., Schussler N., Zaorsky N.G., Jia A.Y., Spratt D.E. (2024). Use of Decipher Prostate Biopsy Test in Patients with Favorable-risk Disease Undergoing Conservative Management or Radical Prostatectomy in the Surveillance, Epidemiology, and End Results Registry. Eur. Urol. Oncol..

[80] Leapman M.S., Sutherland R., Gross C.P., Ma X., Seibert T.M., Cooperberg M.R., Catalona W.J., Loeb S., Schulman-Green D. (2024). Patient experiences with tissue-based genomic testing during active surveillance for prostate cancer. BJUI Compass.

[81] Kornberg Z., Cowan J.E., Westphalen A.C., Cooperberg M.R., Chan J.M., Zhao S., Shinohara K., Carroll P.R. (2019). Genomic Prostate Score, PI-RADS™ version 2 and Progression in Men with Prostate Cancer on Active Surveillance. J. Urol..

[82] Lin D.W., Zheng Y., McKenney J.K., Brown M.D., Lu R., Crager M., Boyer H., Tretiakova M., Brooks J.D., Dash A. (2020). 17-Gene Genomic Prostate Score Test Results in the Canary Prostate Active Surveillance Study (PASS) Cohort. J. Clin. Oncol..

[83] Cedars B.E., Washington S.L., Cowan J.E., Leapman M., Tenggara I., Chan J.M., Cooperberg M.R., Carroll P.R. (2019). Stability of a 17-Gene Genomic Prostate Score in Serial Testing of Men on Active Surveillance for Early Stage Prostate Cancer. J. Urol..

[84] Kaul S., Wojno K.J., Stone S., Evans B., Bernhisel R., Meek S., D’Anna R.E., Ferguson J., Glaser J., Morgan T.M. (2019). Clinical Outcomes in Men with Prostate Cancer who Selected Active Surveillance Using a Clinical Cell Cycle Risk Score. Pers. Med..

[85] Catalona W.J., Partin A.W., Sanda M.G., Wei J.T., Klee G.G., Bangma C.H., Slawin K.M., Marks L.S., Loeb S., Broyles D.L. (2011). A multicenter study of [−2]pro-prostate specific antigen combined with prostate specific antigen and free prostate specific antigen for prostate cancer detection in the 2.0 to 10.0 ng/ml prostate specific antigen range. J. Urol..

[86] Darst B.F., Chou A., Wan P., Pooler L., Sheng X., Vertosick E.A., Conti D.V., Wilkens L.R., Le Marchand L., Vickers A.J. (2020). The Four-Kallikrein Panel Is Effective in Identifying Aggressive Prostate Cancer in a Multiethnic Population. Cancer Epidemiol. Biomark. Prev..

[87] Auvinen A., Tammela T.L.J., Mirtti T., Lilja H., Tolonen T., Kenttämies A., Rinta-Kiikka I., Lehtimäki T., Natunen K., Nevalainen J. (2024). Prostate Cancer Screening with PSA, Kallikrein Panel, and MRI: The ProScreen Randomized Trial. JAMA.

[88] Grönberg H., Eklund M., Picker W., Aly M., Jäderling F., Adolfsson J., Landquist M., Haug E.S., Ström P., Carlsson S. (2018). Prostate Cancer Diagnostics Using a Combination of the Stockholm3 Blood Test and Multiparametric Magnetic Resonance Imaging. Eur. Urol..

[89] Madendere S., Kılıç M., Palaoğlu E., Veznikli M., Vural M., İğdem A., Tilki D., Esen T., Balbay D. (2025). Role of the Stockholm3 Test in Guiding Confirmation Biopsy Decisions for Patients with Prostate Cancer on Active Surveillance. Eur. Urol. Focus..

[90] McKiernan J., Donovan M.J., O’Neill V., Bentink S., Noerholm M., Belzer S., Skog J., Kattan M.W., Partin A., Andriole G. (2016). A Novel Urine Exosome Gene Expression Assay to Predict High-grade Prostate Cancer at Initial Biopsy. JAMA Oncol..

[91] Erdmann K., Distler F., Gräfe S., Kwe J., Erb H.H.H., Fuessel S., Pahernik S., Thomas C., Borkowetz A. (2024). Transcript Markers from Urinary Extracellular Vesicles for Predicting Risk Reclassification of Prostate Cancer Patients on Active Surveillance. Cancers.

[92] Klein E.A., Cooperberg M.R., Magi-Galluzzi C., Simko J.P., Falzarano S.M., Maddala T., Chan J.M., Li J., Cowan J.E., Tsiatis A.C. (2014). A 17-gene Assay to Predict Prostate Cancer Aggressiveness in the Context of Gleason Grade Heterogeneity, Tumor Multifocality, and Biopsy Undersampling. Eur. Urol..

[93] Cuzick J., Swanson G.P., Fisher G., Brothman A.R., Berney D.M., Reid J.E., Mesher D., Speights V.O., Stankiewicz E., Foster C.S. (2011). Prognostic value of an RNA expression signature derived from cell cycle proliferation genes in patients with prostate cancer: A retrospective study. Lancet Oncol..

[94] Cooperberg M.R., Simko J.P., Cowan J.E., Reid J.E., Djalilvand A., Bhatnagar S., Gutin A., Lanchbury J.S., Swanson G.P., Stone S. (2013). Validation of a cell-cycle progression gene panel to improve risk stratification in a contemporary prostatectomy cohort. J. Clin. Oncol..

[95] Cooperberg M.R., Freedland S.J., Schlomm T., Reid J.E., Stone S., Brawer M.K. (2014). Predicting radical prostatectomy outcome: Cell cycle progression (CCP) score compared with primary Gleason grade among men with clinical Gleason less than 7 who are upgraded to Gleason 7. J. Clin. Oncol..

[96] Singh J. (2023). Current and emerging tissue-based molecular biomarkers for prostate cancer management: A narrative review. UroPrecision.

[97] Blume-Jensen P., Berman D.M., Rimm D.L., Shipitsin M., Putzi M., Nifong T.P., Small C., Choudhury S., Capela T., Coupal L. (2015). Development and Clinical Validation of an In Situ Biopsy-Based Multimarker Assay for Risk Stratification in Prostate Cancer. Clin. Cancer Res..

[98] Klein E.A., Haddad Z., Yousefi K., Lam L.L.C., Wang Q., Choeurng V., Palmer-Aronsten B., Buerki C., Davicioni E., Li J. (2016). Decipher Genomic Classifier Measured on Prostate Biopsy Predicts Metastasis Risk. Urology.

[99] Erho N., Crisan A., Vergara I.A., Mitra A.P., Ghadessi M., Buerki C., Bergstralh E.J., Kollmeyer T., Fink S., Haddad Z. (2013). Discovery and Validation of a Prostate Cancer Genomic Classifier that Predicts Early Metastasis Following Radical Prostatectomy. PLoS ONE.

[100] Ross A.E., Loeb S., Landis P., Partin A.W., Epstein J.I., Kettermann A., Feng Z., Carter H.B., Walsh P.C. (2010). Prostate-specific antigen kinetics during follow-up are an unreliable trigger for intervention in a prostate cancer surveillance program. J. Clin. Oncol..

[101] Whitson J.M., Porten S.P., Hilton J.F., Cowan J.E., Perez N., Cooperberg M.R., Greene K.L., Meng M.V., Simko J.P., Shinohara K. (2011). The relationship between prostate specific antigen change and biopsy progression in patients on active surveillance for prostate cancer. J. Urol..

[102] Ng M.K., Van As N., Thomas K., Woode-Amissah R., Horwich A., Huddart R., Khoo V., Thompson A., Dearnaley D., Parker C. (2009). Prostate-specific antigen (PSA) kinetics in untreated, localized prostate cancer: PSA velocity vs PSA doubling time. BJU Int..

[103] Iremashvili V., Manoharan M., Lokeshwar S.D., Rosenberg D.L., Pan D., Soloway M.S. (2013). Comprehensive analysis of post-diagnostic prostate-specific antigen kinetics as predictor of a prostate cancer progression in active surveillance patients. BJU Int..

[104] Aktas S., Yücetaş U., Yücetaş E., Ates H.A., Genc C., Erkan E. (2025). The efficacy of prostate health index (PHI) in predicting pathological progression in low-risk localized prostate cancer cases under active surveillance protocol. World J. Urol..

[105] Hougen H.Y., Reis I.M., Han S., Prakash N.S., Thomas J., Stoyanova R., Castillo R.P., Kryvenko O.N., Ritch C.R., Nahar B. (2025). Evaluating 4Kscore’s role in predicting progression on active surveillance for prostate cancer independently of clinical information and PIRADS score. Prostate Cancer Prostatic Dis..

[106] Gómez-Acebo I., Valero-Dominguez S., Llorca J., Alonso-Molero J., Belmonte T., Castaño-Vinyals G., Molina-Barceló A., Marcos-Gragera R., Kogevinas M., Rodríguez-Cundín P. (2025). Role of circulating MicroRNAs in prostate cancer diagnosis and risk stratification in the MCC Spain study. Sci. Rep..

[107] Liu R.S.C., Olkhov-Mitsel E., Jeyapala R., Zhao F., Commisso K., Klotz L., Loblaw A., Liu S.K., Vesprini D., Fleshner N.E. (2018). Assessment of Serum microRNA Biomarkers to Predict Reclassification of Prostate Cancer in Patients on Active Surveillance. J. Urol..

[108] Hendriks R.J., van der Leest M.M.G., Israël B., Hannink G., YantiSetiasti A., Cornel E.B., Hulsbergen-van de Kaa C.A., Klaver O.S., Sedelaar J.P.M., Van Criekinge W. (2021). Clinical use of the SelectMDx urinary-biomarker test with or without mpMRI in prostate cancer diagnosis: A prospective, multicenter study in biopsy-naïve men. Prostate Cancer Prostatic Dis..

[109] Tosoian J.J., Loeb S., Kettermann A., Landis P., Elliot D.J., Epstein J.I., Partin A.W., Carter H.B., Sokoll L.J. (2010). Accuracy of PCA3 measurement in predicting short-term biopsy progression in an active surveillance program. J. Urol..

[110] Shore N., Concepcion R., Saltzstein D., Lucia M.S., van Breda A., Welbourn W., Lewine N., Gustavsen G., Pothier K., Brawer M.K. (2014). Clinical utility of a biopsy-based cell cycle gene expression assay in localized prostate cancer. Curr. Med. Res. Opin..

[111] Ghabili K., Paulson N., Syed J.S., Nawaf C.B., Khajir G., Martin D.T., Onofrey J., Leapman M.S., Levi A., Weinreb J.C. (2021). Doubling of Decipher Biopsy Genomic Score Is Related to Disease Reclassification on Subsequent Surveillance Biopsy but Not Adverse Features on Radical Prostatectomy. Case Rep. Urol..

