# Supplementary material for: Molecular and Genetic Biomarkers in Prostate Cancer Active Surveillance: Recent Developments and Future Perspectives
Source: Genes (Basel). 2026 Jan 6;17(1):71. doi: 10.3390/genes17010071 (PMC12840713; doi:10.3390/genes17010071)
Supplement: Supplementary file 1 [file genes-17-00071-s001.zip › genes-4067403-supplementary.pdf]

Supplementary Materials

Table S1: Summary of active surveillance cohorts with long-term survival data

Reference	Cohort	Country	Number of patients on AS	Median age	AS inclusion criteria / cohort characteristics	AS protocol	Treatment rate	PCa specific survival	Overall survival
Dall'Era et al, 2008 [15]	University of California-San Francisco (UCSF); Initial cohort	USA	321	63	PSA <10 ng/mL, Gleason sum ≤6, absence of Gleason grade 4 or 5, cancer involvement of <33% of biopsy cores, and clinical T1/T2a tumor.	Office visits with DRE, serial PSA measurements (usually at 3-month intervals), and transrectal ultrasonography (TRUS) at 6-12 month intervals.<br><br>Starting in 2003, repeat prostate biopsies were recommended at 12-24 month intervals for all patients.	24%<br>(3 year)	100%<br>(5 year)	100%<br>(5 year)<br><br>98%<br>(10 year)
Welty et al. 2015 [16]	University of California-San Francisco (UCSF); New cohort	USA	810	62	Low and favourable intermediate-risk PCa<br>PSA ≤10 ng/ml, clinical stage T1/2, biopsy Gleason grade ≤ 3 + 3, ≤ 33% positive cores and ≤50% tumour in any single core	Serum PSA 3 monthly, 6 monthly transrectal ultrasound, annual biopsy.<br><br>The primary trigger for treatment was biopsy reclassification, but additional indications for discussion of treatment were patient anxiety, CAPRA risk re-classification and change in cT stage.	40%<br>(5 year)	100%<br>(5 year)	98%<br>(5 year)
Klotz et al. 2015 [17]	Toronto	Canada	993	68	Low-risk patients (Gleason score ≤ 6 and PSA ≤ 10 ng/mL) and to patients older than age 70 years with PSA ≤ 15 ng/mL or Gleason score ≤ 3+4 until December 1999. Since January 2000, the study was restricted to low-risk patients (Gleason score ≤ 6 and PSA ≤ 10 ng/mL) or patients with favourable intermediate-risk disease (PSA 10-20 ng/mL and/or Gleason score 3+4) with significant comorbidities and a life expectancy of less than 10 years.	Serum PSA every 3 monthly in first 2 years, followed by 6 monthly. Confirmatory biopsy at 12 months, and then every 3-4 years until patient age 80 years.<br><br>PSA kinetics and/or histologic progression were the triggers for intervention initially. After 2009, adverse kinetics were discontinued as the sole trigger for intervention and prompted repeat biopsies or mpMRI.	24.3%<br>(5 year)<br><br>36.5%<br>(10 year)<br><br>45%<br>(15 year)<br><br>45%<br>(20 year)	98.1%<br>(10 year)<br><br>94.3%<br>(15 year)	80%<br>(10 year)<br><br>62%<br>(15 year)
Bokhorst et al. 2016 [18]	Prostate Cancer Research International	International	5302	66	Gleason 3+3, PSA ≤10ng/ml, ≤2 biopsy cores positive for prostate cancer, clinical stage ≤T2c	3 monthly serum PSA and 6 monthly DRE in first 2 years. PSA 6 monthly and DRE yearly thereafter.	52%<br>(5 years)	>99%<br>(5 year)	96%<br>(5 year)

	Active Surveillance study (PRIAS)	(Netherlands, Norway, Japan, Canada, France, Czech Republic, Austria, Italy, Germany, Spain, Turkey, Finland, Greece, Belgium, Switzerland, Sweden, Australia, New Zealand)			Inclusion criteria expanded in 2012 and 2015 to included minimal Gleason 3+4.	Protocolised prostate biopsy at 1, 4, 7 and 10 years post-diagnosis (and subsequently every 5 years). If PSA DT between 0-10, yearly biopsies recommended. Bone scan if PSA≥20ng/ml.<br><br>Switch to active treatment recommended if Gleason >3+3, >2 positive cores and stage >cT2.	73%<br>(10 years)	>99%<br>(10 year)	88%<br>(10 year)
Godtman et al, 2016 [19]	Göteborg	Sweden	474	66	Very low to intermediate risk PSA screen-detected localised PCa.<br><br>Defined as:<br>Very low risk (T1c, not N1 or M1; Gleason score ≤ 6; PSA density < 0.15 ng/ml; < 3 cores with cancer; and ≤ 50% cancer in any core). Low-risk (T1, not N1 or M1; Gleason score ≤ 6; and PSA < 10 ng/ml but not meeting the very low-risk criteria). Intermediate risk (T1-2, not N1 or M1; Gleason score ≤ 7; and/or PSA < 20 ng/ml and not meeting the very low- or low-risk criteria)<br><br>Very low risk: 51% (n=244)<br>Low risk: 27% (n=126)<br>Intermediate risk: 22% (n=104)	PSA 3-6 monthly, re-biopsies if disease progression suspected (PSA and/or cT-stage progression).<br><br>Progression in PSA, histological upgrade at re-biopsy (worse Gleason score or larger cancer volume) and/or clinical progression were triggers for intervention.	53%<br>(10 year)<br><br>53%<br>(15 year)	99.5%<br>(10 year)<br><br>96%<br>(15 year)	80%<br>10 year)<br><br>51%<br>(15 year)

Tosoian, et al. 2020 [20]	Johns Hopkins	USA	1818	66	Very-low-risk and low-risk prostate cancer<br><br>Defined as:<br>Very-low-risk: clinical stage T1c, prostate-specific antigen (PSA) density (PSAD) <0.15 ng/ml, GG1, two or fewer positive biopsy cores, and ≤50% cancer involvement of any biopsy core<br>Low-risk: clinical stage ≤T2a, PSA <10 ng/ml, and GG1	6 monthly serum PSA and DRE, annual prostate biopsy.<br>Definitive treatment recommended to all patients with biopsy grade reclassification GG ≥2	36%<br>(5 year)<br><br>48%<br>(10 year)<br><br>52%<br>(15 year)	99.9%<br>(10 year)	93%<br>(10 year)
Carlsson et al. 2020 [21]	Memorial Sloan Kettering Cancer Centre (MSKCC)	USA	2664	62	Grade Group 1 prostate cancer	6 monthly serum total PSA, 6 monthly DRE and general health review.<br>Non-targeted systematic biopsy generally repeated every 2 to 3 years but not strictly protocolised. More recently, MRI used and change in PSA or MRI prompted biopsy prior to 3-year follow-up.<br>Intervention triggered by patient preference or grade progression (Gleason 4 on biopsy), stage progression on DRE or imaging (cT2c or cT3).	24%<br>(5 year)<br><br>36%<br>(10 year)<br><br>42%<br>(15 year)	100%<br>(10 year)	94%<br>(10 year)
Cooley et al. 2021 [22]	Cooley-Catalona Meta-Dataset	International<br><br>(USA, Canada, Netherlands, Australia)	6775	64	No exclusions<br>Modified NCCN/AUA guidelines risk stratification*:<br>Low risk: 68% (n=4604)<br>Intermediate risk: 19% (n=1288)<br>High risk: 13% (n=882)<br><br>*Low-risk defined as Gleason grade group (GG) 1 (Gleason score 3+3), PSA <10 ng/ml, clinical-stage cT1, and <3 positive biopsy cores. Intermediate-risk patients had any of the following without any high-risk or high-volume criteria: GG2 (Gleason 3+4), PSA 10–20 ng/ml, stage cT2 or 3 positive biopsy cores of any Gleason grade. High-risk patients had any of the following: ≥GG3 (≥Gleason 4+3), PSA ≥20 ng/ml, stage ≥cT3 or ≥4 positive biopsy cores of any GG.	No strict protocol.<br>Reasons for conversion to treatment included grade reclassification, PSA progression, tumour volume progression and anxiety.	33%<br>(6.7 years)	99.5%<br>(6.7 years)	95.5%<br>(6.7 years)

Hamdy et al. 2023 [23]	Prostate Testing for Cancer and Treatment(ProtecT)	UK	545	62	Mostly low-risk PSA screen-detected clinically localised prostate cancer<br><br>CPG 1: 70% (n=382)<br>CPG 2: 21% (n=116)<br>CPG 3-5: 9% (n=47)	"Active monitoring". Serum PSA monitoring every 3 months in first year, every 6 months thereafter. Rise of at least 50% in PSA during previous 12m triggered repeat testing within 6-9 weeks.<br>No protocolised biopsy.<br>If PSA concentrations persistently raised or patient concerns expressed, urologist appointments were made for discussion of further tests (re-biopsy) and all management options.	61% (15 year)	97% (15 year)	77% (15 year)
Herlemann et al. 2024 [24]	Cancer of the Prostate Strategic Urologic Research Endeavor (CaPSURE)	USA	1020	72	Patients managed with either active surveillance or watchful waiting.<br><br>CAPRA risk group<br>Low (0-2): 65% (n=664)<br>Intermediate (3-5): 30% (n=301)<br>High (6-10): 5.4% (n=55)	Not specified	32% (7 years)	Not reported	Not reported
Leclercq et al. 2024 [25]	French Surveillance Active du Cancer de la Prostate (SurACaP) database	France	86	64	SurACaP inclusion criteria:<br>Aged ≤ 75 years and with a life expectancy > 10 years, with clinically localized low-grade PCa (i.e., ISUP 1), clinical stage T1c/T2a, PSA ≤ 10 ng/mL and ≤ 3 positive cores and tumor length ≤ 3 mm per core	3 monthly serum PSA for first 2 years, 6 monthly thereafter. DRE 6 monthly. Confirmatory biopsy in first year, then follow-up biopsy every 2 years or if disease progression suspected.<br><br>Triggers for intervention: PSA progression, grade reclassification at re-biopsy (ISUP ≥ 2), progression in cancer volume or patient request.	78.8% (15 years)	90.9% (15 year)	94.3% (10 year)<br><br>77.5% (15 year)
Newcomb et al. 2024 [26]	Canary Prostate Active Surveillance Study (PASS)	USA	2155	63	Mostly very low or low-risk prostate cancer. No exclusions based on grade group or PSA.<br><br>NCCN risk group:<br>Very low: 41% (n=874)<br>Low: 42% (n=911)<br>Favourable intermediate: 15% (n=319)<br>Unfavourable intermediate: 2% (n=46)<br>High: <1% (n=5)	Serum PSA every 3 months before 2020, and every 6 months from 2020. Prostate biopsies protocol directed at 6 to 12 months after diagnosis, 2 years after diagnosis, and then every 2 years.<br>MRI and biomarker tests were performed at clinician's discretion.	49% (10 year)	99.9% (10 year)	94.3% (10 year)
Selvadurai et al. 2013 [28]	Royal Marsden cohort	UK	471	66	Histologically proven prostate adenocarcinoma, age 50-80 yr, stage T1/T2, prostate-specific antigen level (PSA) <15 ng/ml, Gleason score (GS) ≤ 3+3 (GS ≤ 3+4 if aged >65 yr), and	Patients were assessed by serum PSA level, and digital rectal examination at 3-mo intervals in year 1, 4-mo intervals in year 2, and at 6-mo intervals thereafter.	30% (5 year)	99.9% (5 year)	96% (5 year)

					percent positive biopsy cores (PPC) ≤ 50%.	Transrectal ultrasound-guided prostate biopsy was performed after 18-24 mo and every 2 yr thereafter. Treatment was recommended for PSA velocity (PSAV) >1 ng/ml per year or adverse histology, defined as GS ≥ 4+3 or PPC >50%.			
Tohi et al. 2025 [27]	Global Action Plan (GAP3) international AS consortium	International (USA, Canada, UK, Netherlands, Germany, Ireland, Sweden, Switzerland, Singapore, Australia, Japan, Korea)	24656	65	Localised prostate cancer<br><br>Distribution by Gleason grade group:<br>GG1: 89% (n=21660)<br>GG2: 9.6% (n=2327)<br>GG3: 1.1% (n=275)<br>GG4: 0.2% (n=48)<br>GG5: 0.1% (n=16)	Not reported	Not reported	98.7% (15 years)	88.5% (15 years)
